# Supplementary material for: Differentiation between descending thoracic aortic diseases using machine learning and plasma proteomic signatures
Source: Clin Proteomics. 2024 Jun 2;21:38. doi: 10.1186/s12014-024-09487-4 (PMC11145886; doi:10.1186/s12014-024-09487-4)
Supplement: Supplementary file 1 — Supplementary material 1: Figure S1. Quality control analysis of digestion and mass spectrometry reference plasma pools [file 12014_2024_9487_MOESM1_ESM.docx]

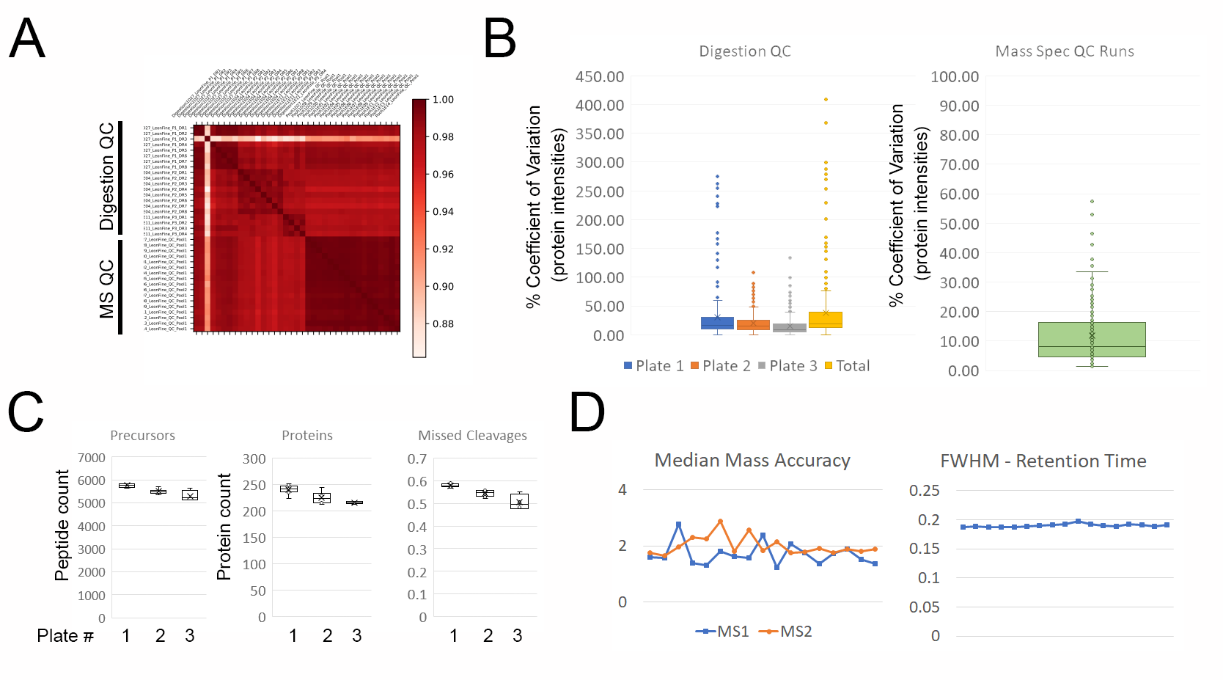


**Supplemental Figure 1**. Quality control analysis of reference plasma pools for monitoring digestion and mass spectrometry performance. **A.** Sample correlations between separate digestions of the same control plasma pool spread throughout all processing plates (digestion QC) and separate mass spectrometry runs on the same pooled digest (MS QC) spread throughout the total acquisition period. **B.** Coefficient of Variation (%CV) for QC samples within and across processing plates (left panel) and MS QC runs (right panel). **C.** Protein and peptide identifications across the processing plates. **D.** Mass accuracy and chromatography performance (full width half max) across all MS QC runs.
